# Factors of Capital on Depression in Older Adulthood: A Comparison of Urban and Rural Regions in Korea

**DOI:** 10.3390/healthcare11212850

**Published:** 2023-10-29

**Authors:** MinYoung Bae, YunYoung Kim, Ijin Hong

**Affiliations:** 1 Department of Social Welfare, Jeonbuk National University, Jeonju 54896, Republic of Korea; 2 Graduate Institute of National Development, National Taiwan University, Taipei 106216, Taiwan; ijinh@ntu.edu.tw

**Keywords:** old age capital, depression, psychosocial environment theory, urban and rural regions

## Abstract

This study analyses old-age capital in its economic, cultural, and social components, in terms of how it impacts on depression in the elderly, comparing urban and rural regions. Our comparative analysis in urban and rural areas focuses on South Korea, using the Korean Welfare Panel Data from 2012 to 2020. Time-series trends and variables measuring capital and depression in older adults were examined in panel data analyses. Depression among the Korean elderly was at a similar level in urban and rural areas, whereas satisfaction regarding income levels, leisure life, and social relationships was higher for older adults in rural areas. We also found that the higher the economic capital, the higher the leisure life satisfaction (cultural capital), and the higher the social relationship satisfaction (social capital), the lower the rates of depression. Finally, depression among the urban elderly did not decrease as house prices increased as a component of economic capital, and depression decreased among groups participating in volunteer activities as part of the social capital of the rural elderly. In accordance with the socioemotional selectivity theory, older adults in rural areas in Korea have an advantage in terms of cultural capital due to their environment, whereas the psychosocial environment theory is relevant to urban elderly people experiencing relative deprivation in terms of economic capital.

## 1. Introduction

The experience of loss in older adulthood, accompanied by the deprivation of financial and social resources, leads to poverty, morbidity, idleness, and isolation, thereby triggering depression [1,2,3], which may even result in suicide in severe cases [4,5,6,7]. While it is difficult to investigate personal and environmental resources systematically, they can be examined based on the three forms of capital proposed by Bourdieu (1986) [1], who defined capital as ‘accumulated labor in its materialized form or incorporated embodied form’ and argued that individuals or groups can own social energy through an accumulation of capital [8,9].

Since the 1960s, South Korea has grown based on an urban-centered industrialization and industrialization strategy, resulting in rural areas being relatively underdeveloped compared to urban areas.

According to the results of the 2022 Agriculture, Forestry and Fisheries Survey released by Statistics Korea (2023), the rural population is approximately 2.17 million, which is less than half of what it was 20 years ago in 2002, when it was approximately 5.22 million [10]. In addition, the proportion of elderly people aged 65 and over in the total population is 49.8%, which is almost 3-fold higher than the 17.5% of the total elderly population in Korea in 2022. Cities are more affluent than rural areas, better supplied with jobs, housing, healthcare, education, and cultural life, but inequality, exclusion from opportunities, and loss of community are more severe in cities than in rural areas. In particular, the population of Korean rural areas is aging, but it has maintained a stronger sense of community through farming based on agglomerated villages compared to cities. Previous studies [11,12,13,14] show that the level of social capital of older adults can vary depending on their level of community and the quality of their social connections.

In Korea, the distribution of capital varies by region, and the mental health of the elderly may also be affected. The rural older population is excluded from economic, educational, and cultural domains compared with their urban counterparts. However, the former’s active community involvement based on solidarity and a sense of community helps reduce their level of depression [15]. Cottam (2011; 2018) stressed the importance of relational welfare and argued that true social welfare involves identifying the necessary competencies for improving individuals’ lives and linking individuals to various resources by fostering good relationships with members of their communities [16,17]. Furthermore, improving and restoring social relationships have been observed to be in line with social movements pursuing health and well-being [17,18].

Therefore, using data from the nationally representative Korean Welfare Panel Study, this work aimed to investigate the factors of changes in geriatric depression over time according to regional resources and present academic and policy implications based on the findings [19].

## 2. Theoretical Background

### 2.1. Capital and Depression

According to German economist Karl Marx (1867), ‘capital is the profit (surplus value) generated through an exchange between production and consumption based on social relationships between capitalists and laborers’ [20]. French sociologist Pierre Bourdieu (1982) [1] divided capital into three forms in the book Forms of Capital: (i) economic capital, which is ‘immediately and directly convertible to money and may be institutionalized in the form of property rights’, some of which are income and assets; (ii) cultural capital, which is classified as ‘embodied’ cultural attitude, activities, and information; (iii) social capital, which refers to trust, norms, reciprocity, network, and social participation derived from social relationships [1,8,19,21]. Bourdieu (1986) argued that these forms of capital may function in different fields, and individuals’ positions may differ depending on their possession of capital useful in each field [1,22]. Depression is a mental disorder that affects people of various ages and is characterized by symptoms such as loss of interest, guilt, diminished self-esteem, and insomnia; severely depressed individuals may even choose to commit suicide [23]. Depression in older adulthood particularly warrants close attention, as the symptoms are difficult to differentiate from those of normal aging, which can delay an accurate diagnosis and treatment and may secondarily lead to suicidal ideation. Regarding the causes of depression, Billings and Moos (1982) proposed a comprehensive framework [7]. based on which they argued that inadequate personal and environmental resources can trigger depression and increase the risk of failure to cope with stress, thereby leading to severe depression accompanied by anxiety and helplessness [7].

Abel and Frohlich (2012) stated that an interaction among economic, cultural, and social capital can have an impact on attaining and/or maintaining good health [24]. Pinxten and Lievens (2014) showed that economic and social capital has a positive effect on both physical and mental health, while cultural capital has a positive effect only on physical health [25]. Moreover, Hanklang et al. (2018) reported individual income, serious economic recession, and social dysfunction as predictors of geriatric depression. In particular, volunteering activities as a means of social participation, a component of social capital, alleviate the adverse outcomes of stress and thus increase life satisfaction while diminishing depression and anxiety [26]. These activities have been found to be more effective in older adults with poor social integration [27,28].

Research on the relationship between diverse capital and the mental health of the elderly is an active area of study [29,30]. Diverse capital refers to the various forms of capital (e.g., social, cultural, and human) that individuals possess and can use to enhance their well-being. Studies have found that older individuals with higher levels of diverse capital tend to have better mental health outcomes, such as lower rates of depression and anxiety. For example, Sato et al. (2022) found that older individuals with strong social networks had better mental health outcomes than those with weaker social networks [31]. Additionally, research has suggested that diverse capital may be an important protective factor against cognitive decline in older adults.

In a study by Sato, K. et al. (2022) of Japanese older people, social cohesion and reciprocity at the individual level and reciprocity at the community level that the older people had before the COVID-19 pandemic showed a reduction in depression [31]. In addition, Miao, J. et al. (2022) showed that Hong Kong’s neighborhood-based senior service promotes opportunities for social activity participation for older adults and activates personal interaction, reducing depression [32]. The higher their socioeconomic status, the greater the reduction.

Thus, mental health in older adulthood is associated with personal factors such as sex, age, and marital status, economic capital such as income and assets, cultural capital such as education and religion, and social capital based on social relationships. It is reported to manifest in interaction with the region’s environment [15,33].

### 2.2. Capital and Depression in Older Adulthood and Regions

First, regarding economic capital, Scott and Storper (2003) stated that varying levels of income inequality are witnessed across regions [34]. In particular, globalization and rapid technological advances have had a more tremendous impact on urban or small- and medium-sized cities than large cities [35]. Several studies have reported a consistent correlation between city size and income inequality [36,37]. Furthermore, Marmot and Wilkinson (2001) reported that residents of regions with high income inequality suffer from greater relative deprivation [38]. In this context, the psychosocial environment theory stipulates that relative income, as opposed to absolute income, is directly linked to adults’ health [3].

Bourdieu (1982) discussed cultural capital as a mechanism of ‘embodied’ class reproduction. Thus, in earlier times, cultural capital was discussed based on educational level or occupation [1,39]. However, De Graaf et al. (2000) estimated cultural capital through cultural and leisure activities [40]. According to Havighurst and Alberecht (1953), older adults have social and psychological needs; in general, greater involvement in social activities is linked to a higher quality of life [41]. The World Health Organization (2002) surmised that individuals accumulate cultural capital under the influence of their living environment [42]. This view follows the socioemotional selectivity theory that emphasizes the importance of cultural and social capital in enhancing the quality of life of older adults [2]. Thus, this theory is associated with cultural and social capital.

Regarding social capital, many studies reported that social bonding can affect the level of depression in adults. Moreover, these studies explained that people with stronger social bonding could attain emotional stability even amid stress because they feel supported by others [43,44,45]. Kobayashi et al. (2015) reported that mistrust in the community and lack of interaction intensify older adults’ psychological distress—depression [46]. Kim et al. (2021) reported that rural older adults in Korea display a stronger solidarity and sense of community compared with their urban counterparts and that accumulation of social capital is connected to lower depression [15]. Wang et al. (2019) stated that social capital increases the awareness of healthy behaviors and lowers the cost of searching for health-related information, thereby positively impacting mental health among rural older adults [33]. It particularly improves the mental health of female older adults and those with low income.

As above, existing studies tend to equate environmental factors with urban–rural comparison. In previous studies, the social capital of older adults generally means charitable donations or voluntary work. Several previous studies have found that environmental factors influence older people’s social participation [47,48,49]. In other words, social capital is abundant where there are facilities for the ageing and transportation is suitable. Richard et al., 2008 have demonstrated that the social capital of ageing people in cities is greater than that of those in rural areas. This is because large cities have better infrastructure and transportation than rural areas [49].

**Figure 1:**
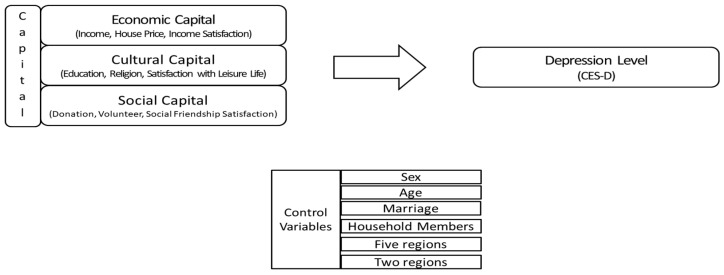
Research framework.

In addition, the main argument in Billings & Moos (1982), Lam et al. (2020) and Shu-Lin Shi (2020) is that environmental resources such as neighborhood attributes play a significant role in determining an individual’s health and well-being [7,50,51]. Kim et al. (2021) also explore the impact of the proximity of resources available in the area where older adults reside on depression and identify the resources that play an important role in reducing or preventing depression among older adults in a Korean context [15]. The relationship between urban–rural disparity and mental health also represents an important research topic in China. According to Zhang (2018) and Li (2021), informal family care is very important in determining the physical and mental health of the elderly, especially for those residing in rural areas [52,53]. The presence of more acute physical and mental health needs in rural areas in China is strongly determined by the accessibility of medical services as well [54]. Therefore, this study aims to examine the effect of capital on depression among the elderly in Korea (see Figure 1).

## 3. Materials and Methods

### 3.1. Data

In this study, data for older adults aged 65 and above from the 7th (2012) to the 15th (2020) Korea Welfare Panel Study were analyzed [19]. The Korea Welfare Panel (hereinafter referred to as the “KoWePS”) has been produced every year since 2006 and surveys more than 7000 households (over 15,000 household members). It identifies the living conditions and welfare needs of various population groups according to age, income and economic activity, and evaluates the effectiveness of policy implementation. The KoWePS samples 517 enumeration districts with 90% data from the Population Census and investigates the household income and economic activity status of the members of the household. A total of 7000 households, comprising 3500 general households and 3500 low-income households, are selected through two-stage stratified sampling. Although the welfare panel study was initiated in 2006, in the 7th survey, new households were added to maintain the initial sample size. In this study, the initial year of analysis was set to 2012.

### 3.2. Study Parameters and Instruments: Geriatric Depression

In this study, geriatric depression was defined as a psychological disorder affecting older adults, characterized by negative emotions such as helplessness, worry, concern, nervousness, and unhappiness, along with cognitive decline and reduced physical activity. The KoWePS uses the 11 items of the Center for Epidemiologic Studies-Depression Scale (CES-D) to measure depression. This tool is an abbreviated and restructured version of the 20-item depression scale developed by Radloff (1977) [55]. It comprises somatic problems (2 items), depressed affect (4 items), positive affect (2 items), and interpersonal failure (3 items); a higher score indicates a higher level of depression. In this study, the scores were converted to the original 20-item scale score by multiplying the total score by 20/11. Based on the original scoring criteria, a total score of ≥16 indicated depression. The Cronbach’s alpha value of the depression variables is 0.8780. Reliability between items was confirmed to be high.

### 3.3. Capital

#### 3.3.1. Economic Capital

In this study, economic capital was defined as individuals’ income and assets that can be ‘immediately and directly converted to money and may be institutionalized in the form of property rights’ based on the definitions presented by Marx (1867) and Bourdieu (1986) [1,20]. With reference to Caro et al. (2014) and Lissitsa (2015), ‘income’, ‘house price’ (a factor that has become increasingly important in Korea in recent years), and ‘satisfaction with household income’ were examined in this study (see Table 1) [56,57].

#### 3.3.2. Cultural Capital

Cultural capital was defined as capital that exists in ‘embodied’, ‘objectified’, and ‘institutionalized’ forms and is institutionalized in the form of educational attributes, based on the definition by Bourdieu (1986) [1]. With reference to Holt (1998) and Tang (2006), ‘educational level’, ‘religion’, and ‘satisfaction with leisure life’ were examined in this study [39,58]. Here, the presence or absence of religion rather than the type of religion was constructed as a measurement index of cultural capital. This is because people with religion can form their own values or ethical resources according to strict rules and norms within religion, and it is thought that this is embodied cultural capital, a sub-factor of cultural capital [59].

#### 3.3.3. Social Capital

Social capital was defined as ‘resources embedded in social networks accessed and used by actors for benefits’ based on the definitions proposed by Bourdieu (1986) and Lin (2001) [1,8]. Further, ‘satisfaction with social relationships’, ‘donation’, and ‘volunteering’ were examined in this study with reference to the following: the US Social Capital Community Benchmark Survey (SCCBS), the UK Social Capital Harmonised Question Set (HQS), social capital-related indices in the World Value Survey (WVS), and Inouye (2007) (see Table 1) [60,61,62,63].

**Table 1:** Variables of capital.

Type of Capital	Authors (Year)	Variable Composition
Economic capital	Caro et al. (2014) [56]	Income and assets, parents’ education level, parents’ employment status
	Lissitsa (2015) [57]	Individual income, social benefits at work
Cultural capital	Holt (1998) [39]	Parents’ education level and occupation, individual’s education level and occupation
	Tang (2006) [58]	Religion, frequency of participation in religious activities, pleasure reading, quality of life
Social capital	Inouye (2007) [63]	Network, reciprocity norms, trust, participation opportunity, formal and informal interaction, life as a member of a community
	SCCBS	Trust (social trust, interracial trust), diversity of friendship, political participation (electoral political participation, participation in protest politics), civil leadership and associational involvement, donation and volunteering, faith-based engagement, equality of civic engagement
	HQS	Social participation, social network and social support, reciprocity and trust, civic engagement, views about community
	Social capital-related indices in the WVS	Social participation, reciprocity, political interest, trust

#### 3.3.4. Other Variables

In this study, the demographic characteristics previously identified as predictors of geriatric depression—gender, age, marital status, and household size—were taken as the control variables.

As shown in Table 2, The Korea Welfare Panel provides five regional variables: 1 to 3 are classified as cities, and 4 and 5 are classified as rural areas. Regional scales were classified according to administrative standards such as large cities, small- and medium-sized cities (=si), and rural areas (=county). Based on the KOSIS population census “https://kosis.kr/index/index.do (accessed on 1 August 2021)”, a region was classified as rural if it was home to fewer than 100,000 people; small- and medium-sized cities are those with 100,000 to 200,000 people; and large cities are those with more than 200,000 people. Additionally, more important than absolute population numbers, the distinction between urban and rural areas (two regions) is centered on the proportion of the agricultural population and cultivated land. The Korean Welfare Panel data provide this regional classification variable, so we have included it in our analysis.

#### 3.3.5. Panel Analysis

First, the trends of the major variables of capital and depression in older adults over time were examined. Subsequently, the panel data with the key independent variables (capital and sociodemographic characteristics) were established and analyzed to identify the predictors of geriatric depression.

An important criterion for panel models is the inferences drawn from the characteristics of the panel objects (μi). If panel data are of a randomly drawn sample from a population, then the error term can assume a probability distribution. The panel analysis procedure includes both random-effects and fixed-effects models, and then the appropriate model must be selected. The Korean Welfare Panel data analyzed in this study consist of a randomly selected sample from the entire population. In this case, it is reasonable to use a random-effects model to estimate variables that do not vary over time, such as religion, education, gender, and region, which are used as the main variables in this study. The estimation of random-effects models has been used to analyze regional comparisons using the Korean National Welfare Panel Data in previous studies as well [15,64]. This is because it is not possible to estimate time-invariant variables such as gender and education by estimating fixed-effects models.

Therefore, the reasons for using a random-effects model can be summarized as follows: (i) identify the time-invariant variables (sex, education, etc.) estimates and (ii) adequately control for time-invariant individual factors to satisfy the assumption of uncorrelated heterogeneity.

This can be estimated with a panel model as follows:

$$y_{it}^{*}=\alpha +\beta x_{it}+\mu_{i}+e_{it}\;\mathrm{If},\;e_{it}=ii+et+\sim 0\;it,\;i\left(individual\right)=1,2\ldots .N,\;t\left(year\right)=1,2\ldots .T$$

μi = unobservable individual effect

it = unobservable time effect

e_it_ = remainder stochastic disturbance term.

## 4. Analysis Results

Table 3 below shows the general characteristics and study variables of the participants as recorded in the 2012–2020 KoWePS surveys. First, approximately 4000–5300 participants were surveyed every year from 2012 to 2020, with approximately 38,000–45,000 participants being surveyed for nine years. The mean depression score was 9.54, with a standard deviation (SD) of 9.69. The mean scores for satisfaction with income, leisure life, and social relationships were 2.79, 3.25, and 3.63, respectively.

**Figure 2:**
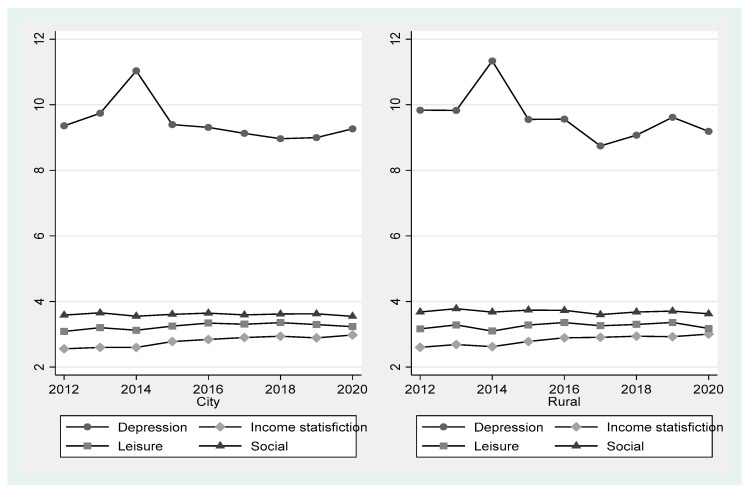
Descriptive statistics of major study variables.

A time-series comparison of urban and rural regions is shown in Figure 2. The depression score was the highest in 2014 for both urban and rural regions, at 11.03 and 11.34, respectively; in 2020, the scores were 9.26 and 9.19, respectively. The variations and deviations of the scores were greater in the rural regions. The mean scores for satisfaction with income, leisure life, and social relationships among older adults were 2.79, 3.25, and 3.6, respectively, in urban regions and 2.82, 3.26, and 3.69, respectively, in rural regions. Thus, the rural regions showed slightly higher scores.

Urban areas in Korea are highly agglomerated due to a large population and high population density. Since several residents join the metropolitan areas due to more employment opportunities, these environments remain rather impersonal, and local community ties are not easily formed. On the other hand, residents in rural areas have an advantage in developing a sense of community, which may explain why the social satisfaction of the elderly is generally better in rural areas.

Table 4 shows the results of the panel regression analysis with the random-effects model to examine the effects of capital on mental health in older adults. Model 1 is an uncontrolled model without controlling for regions; in Model 2, the rural/urban variable has been added to the analysis. Model 3 compares the degree of depression between regions (compared with the city of Seoul).

In all models, economic variables were closely linked to depression. In other words, depression decreased with increasing disposable income, increasing house prices, and increasing income satisfaction. This may be relevant to the characteristics of the KoWePS. As the KoWePS sample comprises 50% low-income and 50% regular households, even with weight adjustment, the sample cannot be fully controlled; most economic capital variables were found to affect depression. In terms of cultural capital, depression decreased with increasing leisure life satisfaction, while religion and education did not have an impact on depression. In terms of social capital, depression was not significantly associated with donation/volunteering, but it decreased with increasing social relationship satisfaction. In other words, life satisfaction and mental health were generally strongly associated. In terms of the control variables, depression was significantly higher among women (than men), older individuals, and those without a spouse; moreover, depression increased with decreasing household size.

In Model 3, which included the regional variable, older adults living in a metropolitan city, small- or medium-sized city, or rural region had better mental health compared with those living in Seoul. This indicates that perceived happiness is lower among Seoul residents due to the large gap between the rich and poor, which results in urban poverty issues and high relative deprivation.

Table 5 lists the predictors of possession of capital and depression in older adults by urban and rural regions. These results are similar to the previous findings, except that the negative association between house price and depression was not statistically significant in the urban region. This may be because house prices in urban regions vary widely and result in greater relative deprivation. Moreover, depression decreased with increasing household size in urban regions. According to Castells-Quintana et al. (2020), Glaeser et al. (2015), and Storper (2013), economic inequality varies according to the size of a rural or urban region. Therefore, it can be speculated that house prices influenced older adults’ mental health in the present analysis [35,36,37].

In the 2018 KoWePS survey, the Gini coefficient was 0.3 in large cities and 0.28 in rural agricultural regions as shown in Table 6. Additionally, the house price is per household. However, in Korea, house prices and living conditions tend to be very closely linked. Therefore, even if it is not possible to know exactly whether or not the house is owned by those living in it, it seems that there is a basis for presuming that the residential environment is good if the house price is high. Korea has a low population density and is sensitive to housing prices, and the better the environment, the higher the house price. This is also substantiated by data from the National Statistical Office of Korea.

Three types of satisfaction and income significantly predicted mental health in rural and urban regions, but cultural capital did not have a marked effect on mental health. In essence, depression was lower in the group with two forms of social capital—engagement in volunteering and donation—only in the rural regions. This is consistent with the results of De Graaf et al. (2000) and Fung et al. (2008), who found that social engagement was beneficial for mental health in rural regions, where community activities are organized frequently and vigorously [2,40].

## 5. Discussion

Existing studies on mental health among older adults have primarily focused on individual characteristics, such as age, sex, educational level, and household type, or simply emphasized the importance of a particular form of capital [26,65]. As a result of referring to such fragmentary research data, mental health policies in Korea have been limited. Therefore, this study conceptualized the possession of capital in older adulthood in terms of economic, cultural, and social capital in Korea. This study examined whether these three forms of capital are negatively associated with depression as demonstrated by Abel and Frohlich (2012) and Pinxten and Lievens (2014) [24,25].

Abel and Frohlich (2012) argued that disparities in economic, cultural, and social capital engender health disparity [24]. Pinxten and Lievens (2014) showed that groups with stronger social cohesion, such as those with higher education, large economic capital, and neighbors, displayed better mental health [25]. Our findings are in line with these results to some extent. However, our sample was limited to older adults; thus, the gap in education level was not as large as that among the younger generations. This may have contributed to the lack of a significant effect of education on mental health.

Thus, unlike rural regions, house prices in urban regions did not reduce the depression score. Moreover, the scores for depression were generally lower in the small- and medium-sized cities and rural regions compared with Seoul. In light of Wilkinson’s (1996) psychosocial environment theory, the present findings suggest that older adults’ relative income affects their mental health [3].

However, older adults are bound to be heavily influenced by their environment as they experience significant changes in the range of their physical movement and social relationships after retirement due to diminished physical functioning and financial power [66]. At least in this study, social engagement had a significant impact on older adults’ mental health, and older adults in rural regions were at an advantage in terms of community-based social engagement over their urban counterparts.

Specifically, two components of social capital—donation and volunteering—were found to reduce depression in rural older adults, suggesting that active aging boosts their life satisfaction and lowers depression [42]. This can be discussed in continuum with the findings of Fengler (1984) and Musick et al. (1999), where such effects were more evident among older adults with few resources and social interactions [67,68]. Therefore, policies that can actively identify and enhance the competencies of marginalized older adults in Korea with little existing capital need to be formulated.

In conclusion, the rural older population in Korea can be explained through the socioemotional selectivity theory, while the urban older population can be more effectively explained through the psychosocial environment theory due to their relative deprivation [2,3]. A key implication of this study is that varying forms of capital can explain mental health among older adults depending on the region. Thus, more specific and diverse policies that stress the relational aspect of older adults regardless of class should be implemented in urban regions.

## 6. Conclusions

This study verified the difference between urban and rural areas in terms of the effect of capital on depression among the elderly in Korea. First of all, the factors that commonly affect depression in urban and rural elderly were income, income satisfaction, leisure life satisfaction, and social relationship satisfaction, and as these factors increased, the level of depression decreased. Secondly, in the case of the urban elderly, as the number of family members living together increased, the level of depression decreased. House prices did not affect the level of depression. In addition, in the case of rural elderly, as volunteering and donation activities increased, the level of depression decreased.

Currently, as the number of elderly people living alone is rapidly increasing in Korea, there are also many lonely deaths [69]. Although the number of elderly people living alone is higher in rural areas than in cities, the number of family members living together has a significant effect on the level of depression because rural elderly people have a high sense of community, centered on the village and have strong ties to their neighbors [51,70]. However, in the case of the elderly living alone in the city, it is highly likely that the number of family members living together had an effect on the level of depression, as the sense of community was lower and the neighborhood bonds were weaker than those in the countryside [71]. Several studies have shown that social capital has a positive impact on the sense of community, which not only increases life satisfaction but also reduces depression [70,71,72,73].

Fougeyrollas et al. (1998) and Demers et al. (2009) suggest that environmental factors may be more important than personal characteristics such as gender, age, income, and education in determining the social capital of older adults. Age-friendly city initiatives, which are gaining popularity around the world, are in line with these concerns [74,75]. This study suggests that an age-friendly city should be built based on a network of social relationships for older adults who might otherwise be isolated from society, with the aim to strengthen their sense of community and cohesion with their neighbors, thereby reducing depression. In this respect, an interesting example could be the Global Network of Age-Friendly Cities & Communities (GNAFCC), a global project promoted by the World Health Organization (WHO) since 2006 to effectively respond to the trends of aging and urbanization. As of December 2022, 1445 cities in 51 countries have joined and are interacting with each other (40 local governments in Korea have joined). Among the main areas of Age-Friendly City, social participation, civic participation and employment, community support and health services are closely related to social capital and they may alleviate elderly depression by revitalizing communities and improving social relationship satisfaction. This policy initiative takes into account the social, economic, and cultural contexts of South Korea, as shown in Table 3 and Table 6 [15,64].

In addition, in the case of rural elderly, the higher the income and income satisfaction, the lower the level of depression, and the greater the volunteering and donation activities, the lower the level of depression. Therefore, the basic pension should be raised step by step, and a stable old-age income should be guaranteed by developing a job program for the elderly (suitable for their age and capacity) and raising benefits. In addition, by providing various voluntary and/or donation activities, the social activities of the elderly should be encouraged, and the level of depression should be reduced by increasing their self-efficacy.

We acknowledge that this study suffers from some limitations. First, social capital is defined operationally for statistical analysis, leaving out specific contexts. This is a challenge for future research using qualitative methods. Especially, we leave it to future research to explore the differences in social capital and the means by which to enable it, taking into account the demographic and sociostructural contexts of urban and rural areas.

Additionally, the influence of comorbidities (chronic diseases and disability) was not sufficiently considered when assessing mental health in the study participants. However, the Korea Welfare Panel is reliable and extensive data produced by the Korean government. Therefore, this is more of a fundamental limitation of quantitative research indicators than a problem of this specific data set. These limitations need to be supplemented through follow-up qualitative studies in the future, since pre-existing pathologies and related co-morbidities are likely to be associated with depressive symptoms. Additionally, this study’s findings may find limited generalizability to other countries or regions as they are closely tied to the specific cultural and societal context of Korea. Finally, our findings apply to the older population only. Future research could investigate whether analogous findings can be observed for a more diverse sample population.

## Figures and Tables

**Table 2:** Variables and values used in the analysis.

**Variable**	**Categories**	**Explanation**
Dependent variables	Depression level (CES-D)	
	Questions: I did not feel like eating; my appetite was poor; I felt I was just as good as other people; I felt depressed; I had trouble keeping my mind on what I was doing; My sleep was restless; I felt lonely; I was happy; People were unfriendly; I felt sad; I felt that people disliked me; I could not “get going”	Rarely or none of the time (less than 1 day) = 0, some or a little of the time (1–2 days) = 1, occasionally or a moderate amount of time (3–4 days) = 2, most or all of the time (5–7 days) = 3
Independent variables	Economic capital	
	Income	Disposable income (Log function)
	House price	House price (Log function)
	Income satisfaction	Very Dissatisfied = 0, Dissatisfied = 1, Moderate = 2, Satisfied = 3, Very Satisfied = 4
	Cultural capital	
	Education	Less than high school graduate = 1, High school graduate or higher = 2
	Religion	Religious = 1, No Religion = 2
	Satisfaction with leisure life	Very Dissatisfied = 0, Dissatisfied = 1, Moderate = 2, Satisfied = 3, Very Satisfied = 4
	Social capital	
	Donation Volunteer	no = 1, yes = 2
	Social Friendship Satisfaction	Very Dissatisfied = 0, Dissatisfied = 1, Moderate = 2, Satisfied = 3, Very Satisfied = 4
Control variables		
	Sex	Man = 0, Woman = 1
	Age	65–69 = 0, 70–74 = 1, 75–79 = 2, Oer 80 years old = 3
	Marriage	No = 0, Yes = 1
	Household members	persons
	Five regions	Seoul = 1, area near Seoul = 2, City = 3, rural county = 4, complex county = 5
	Two regions	City = 1 (Add 1–3 above), rural = 2 (Add 4–5 above)

**Table 3:** Descriptive statistical analysis of major variables.

	2012–2020			2012			2020		
Variable	Obs	Mean	S.D	Obs	Mean	S.D	Obs	Mean	S.D
Depression	42,332	9.54	9.69	4945	9.51	9.22	4356	9.24	9.32
lncome	45,013	7.47	0.74	5295	7.33	0.74	4644	7.62	0.71
House	38,006	8.76	1.43	4473	8.58	1.40	3924	9.01	1.44
Satisfaction of income	42,333	2.79	0.87	4946	2.57	0.86	4356	2.99	0.81
**Religion**	**40,481**	**0.58**	**0.49**	**5308**	**0.61**	**0.49**	**4817**	**0.565**	**0.49**
**Education**	**45,144**	**1.18**	**0.39**	**5308**	**1.17**	**0.37**	**4663**	**1.22**	**0.41**
Satisfaction with leisure life	42,331	3.25	0.77	4945	30.11	0.78	4356	3.22	0.74
**Volunteer**	**43,429**	**0.033**	**0.18**	**5103**	**0.028**	**0.17**	**4476**	**0.034**	**0.18**
**Donation**	**45,144**	**1.98**	**0.14**	**5308**	**1.99**	**0.12**	**4663**	**1.98**	**0.15**
Social Friendship satisfaction	42,333	3.63	0.66	4946	3.62	0.67	4356	3.57	0.65
**Sex**	**45,144**	**1.62**	**0.49**	**5308**	**1.60**	**0.49**	**4663**	**1.62**	**0.49**
Age	45,144	75.67	6.66	5308	74.33	6.28	4663	76.86	7.04
**Marriage**	**45,144**	**1.42**	**0.49**	**5308**	**1.40**	**0.49**	**4663**	**1.43**	**0.50**
Household members	45,129	2.02	1.03	5308	2.11	1.12	4662	1.91	0.90

**Table 4:** Factors affecting the mental health of the elderly according to region.

	No Region Variables	Urban/Rural Dummy Variables	Five Regional Dummy Variables Variables
Variables	Depression	Depression	Depression
Income	−0.863 ***	−0.862 ***	−0.872 ***
	(0.11)	(0.11)	(0.11)
House prices	−8.74 × 10^6^ **	−9.27 × 10^6^ **	−1.23 × 10^5^ ***
	(0.00)	(0.00)	(0.00)
Income satisfaction	−1.531 ***	−1.529 ***	−1.525 ***
	(0.06)	(0.06)	(0.06)
Religion	−0.109	−0.111	−0.0944
	(0.11)	(0.11)	(0.11)
Education	−0.265	−0.282	−0.285
	(0.20)	(0.20)	(0.20)
Satisfaction with leisure life	−1.822 ***	−1.822 ***	−1.819 ***
	(0.07)	(0.07)	(0.07)
Volunteering	−0.385	−0.381	−0.395
	(0.41)	(0.41)	(0.41)
Donations	−0.143	−0.140	−0.164
	(0.51)	(0.51)	(0.51)
Social friendship satisfaction	−1.520 ***	−1.516 ***	−1.523 ***
	(0.08)	(0.08)	(0.08)
Sex	2.189 ***	2.187 ***	2.182 ***
	(0.16)	(0.16)	(0.16)
Age	0.176 ***	0.177 ***	0.175 ***
	(0.01)	(0.01)	(0.01)
Marriage	1.371 ***	1.362 ***	1.351 ***
	(0.16)	(0.16)	(0.16)
Household members	−0.218 *** (0.08)	−0.220 *** (0.08)	−0.223 *** (0.08)
D_Areas near Seoul D_City D_Rural county D_Urban−rural complex		0.144 (0.16)	−1.009 *** (0.24) −0.475 ** (0.23) −0.739 *** (0.25) −0.750 * (0.43)
Constant	17.35 ***	17.20 ***	18.18 ***
	(1.27)	(1.28)	(1.29)
Observations	31,852	31,852	31,852
Number of pid	5962	5962	5962

Note: Standard errors in parentheses. *** *p* < 0.01, ** *p* < 0.05, * *p* < 0.1.

**Table 5:** Factors affecting the mental health of the elderly according to urban and rural regions.

	Urban	Rural
Variables	Depression	Depression
Income	−0.889 ***	−0.711 ***
	(0.13)	(0.20)
House prices	−7.16 × 10^6^ *	−2.94 × 10^5^ **
	(0.00)	(0.00)
Income satisfaction	−1.603 ***	−1.313 ***
	(0.07)	(0.11)
Religion	−0.182	−0.0152
	(0.13)	(0.21)
Education	−0.145	−0.742 *
	(0.22)	(0.45)
Satisfaction with leisure life	−1.958 ***	−1.457 ***
	(0.08)	(0.13)
Volunteering	0.0933	−1.555 **
	(0.49)	(0.77)
Donations	0.448	−1.850 **
	(0.59)	(1.03)
Social friendship satisfaction	−1.535 ***	−1.405 ***
	(0.09)	(0.16)
Sex	2.257 ***	2.101 ***
	(0.19)	(0.30)
Age	0.193 ***	0.135 ***
	(0.01)	(0.02)
Marriage	1.419 ***	1.230 ***
	(0.19)	(0.30)
Household members	−0.232 **	−0.150
	(0.09)	(0.15)
Constant	16.42 ***	18.85 ***
	(1.49)	(2.48)
Observations	23,145	8707
Number of pid	4359	1769

Note: Standard errors in parentheses. *** *p* < 0.01, ** *p* < 0.05, * *p* < 0.1.

**Table 6:** The 2015–2019 Gini coefficient by region.

Region	2015	2016	2017	2018	2019
All	0.314	0.308	0.303	0.294	0.297
Seoul/metropolis	0.298	0.291	0.310	0.300	0.302
Rural areas	0.280	0.257	0.298	0.276	0.278

Source: KoWePS (2022). Korea Welfare Panel Study data calculation. https://www.koweps.re.kr.442/data/data/list.do (accessed on 1 August 2021) [19].

## Data Availability

Korean welfare panel data sources: https://www.koweps.re.kr:442/main.do;jsessionid=71381BDC4B427E6842ED7BBB93A35FD5 (accessed on 1 August 2021).
